# Lipid nanodiscs spontaneously formed by an amphiphilic polymethacrylate derivative as an efficient nanocarrier for molecular delivery to intact cells†

**DOI:** 10.1039/d3ra07481a

**Published:** 2024-02-19

**Authors:** Jinyu Hao, Mika Ishihara, Gwénaël Rapenne, Kazuma Yasuhara

**Affiliations:** a Division of Materials Science, Graduate School of Science and Technology, Nara Institute of Science and Technology (NAIST) 8916-5 Takayama-cho Ikoma 630-0192 Japan yasuhara@ms.naist.jp; b CEMES-CNRS, Université de Toulouse, CNRS 29 Rue Marvig F-31055 Toulouse Cedex 4 France; c Centre for Digital Green-innovation, Nara Institute of Science and Technology (NAIST) 8916-5 Takayama-cho Ikoma 630-0192 Japan

## Abstract

There is a great demand for the technology of molecular delivery into living cells using nanocarriers to realise molecular therapies such as gene delivery and drug delivery systems. Lipid-based nanocarriers offer several advantages for molecular delivery in biological systems, such as easy preparation, high encapsulation efficiency of water-insoluble drug molecules, and excellent biocompatibility. In this paper, we first report the interaction of lipid nanodiscs spontaneously formed by the complexation of an amphiphilic polymethacrylate derivative and phospholipid with intact cells. We evaluated the internalisation of polymethacrylate-based lipid nanodiscs by intact HeLa cells and applied them to the delivery of paclitaxel (PTX), an anticancer drug. The lipid nanodisc showed excellent uptake efficiency compared to conventional liposomes at a concentration where nanodiscs do not show cytotoxicity. In addition, the nanodisc encapsulating PTX showed significantly higher anticancer activity than PTX-loaded liposomes against HeLa cells, reflecting their excellent activity in delivering payloads to intact cells. This study demonstrated the potential of a polymethacrylate-based lipid nanodisc as a novel nanocarrier for molecular delivery to intact cells.

## Abstract 2

Lipid nanodisc is a promising nano-carrier for molecular delivery that efficiently delivers hydrophobic molecules to intact cells.

## Graphical abstract

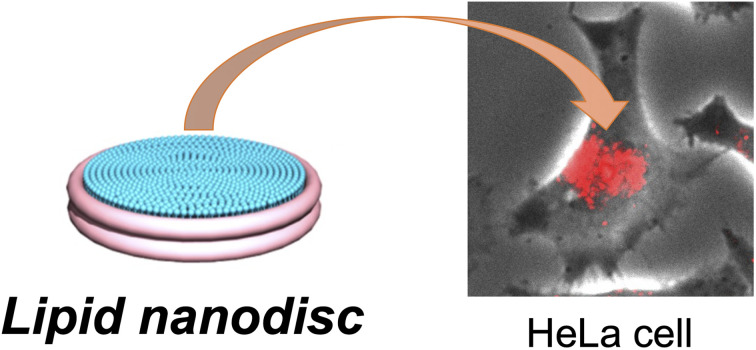

## 1 Introduction

Molecular delivery into living cells using nanocarriers is a pivotal technology in molecular therapies such as gene delivery and drug delivery systems.^1,2^ Various materials have been utilised so far to formulate nanocarriers for molecular delivery including surfactants,^3^ polymers,^4,5^ and inorganic nanoparticles.^6^ Lipid-based nanocarriers have gained significant attention as they offer several advantages such as facile preparation, high encapsulation efficiency of water-insoluble drug molecules, excellent biocompatibility, and low cytotoxicity.^7^ Furthermore, chemical modification of lipid-based nanocarriers allows precise control of membrane properties and functions, realising specific targeting^8^ and stimuli-responsive release of entrapped drug molecules.^9^ Due to their potential in molecular therapies, several nanomedicines employing lipid-based nanocarriers have been already approved for clinical usage.^10^

A lipid nanodisc, which is a class of disc-shaped molecular assembly encompassing the smallest lipid bilayer, is one of the promising lipid-based nanocarrier.^11^ Compared to liposomes, the most typical lipid aggregates in water, nanodiscs have unique characteristics such as their uniform size, size tunability and a large specific surface area. Due to its discoidal shape, lipid nanodisc offers several pharmacological benefits such as improved half-life^12^ and excellent cell internalisation^13^ of drugs compared to conventional lipid-based spherical nanocarriers like liposomes. Several agents such as surfactants,^14,15^ short-chain lipids,^16,17^ membrane scaffold proteins (MSPs)^18,19^ and synthetic polymers^20–22^ are known to form lipid nanodiscs by the complexation with phospholipid membranes. We have previously developed an amphiphilic polymethacrylate random copolymer capable of the spontaneous formation of nanodiscs through the fragmentation of the lipid membrane.^20^ This polymer can produce homogeneous nanodiscs and their size can be tuned by simply changing the polymer-to-lipid mixing ratio. As a biological activity of polymethacrylate-based lipid nanodiscs, we have found that the amyloidogenic proteins such as human islet amyloid polypeptide^20^ and amyloid-β^23^ actively bind to the nanodiscs, resulting in the inhibition of fibril formation and the reduction of their cytotoxicity. While these amyloid-related studies have demonstrated the partial potential of methacrylate-based nanodiscs as biomaterials, the interaction of the nanodisc with intact cells has not been elucidated yet. In this study, we have investigated the internalisation of polymethacrylate-based lipid nanodiscs by intact cells for the first time. In addition, the lipid nanodiscs were used to deliver the anticancer drug paclitaxel (PTX) to cancer cells to demonstrate the potential of polymethacrylate-based lipid nanodiscs as a novel nanocarrier for molecular delivery to living cells.

## 2 Experimental

### Materials

1,2-dipalmitoyl-*sn*-glycero-3-phosphocholine (DPPC) was purchased from the NOF Co. (Tokyo, Japan). Methacroylcholine chloride (80 wt% in H_2_O) was purchased from Sigma-Aldrich Inc. (St. Louis, MO, USA). Butyl methacrylate was purchased from TCI Co., Ltd (Tokyo, Japan). Lissamine rhodamine B 1,2-dihexadecanoyl-*sn*-glycero-3-phosphoethanolamine (Rh-DHPE) was purchased from Thermo Fisher Scientific (Waltham, MA, USA). Cell counting Kit-8 (CCK-8) was purchased from Dojindo (Kumamoto, Japan). PTX was purchased from BLDpharm (Shanghai, China). All other chemicals were purchased from FUJIFILM Wako Pure Chemical Co. (Osaka, Japan) and used without further purification.

### Synthesis of nanodisc-forming polymer

**Scheme 1:**
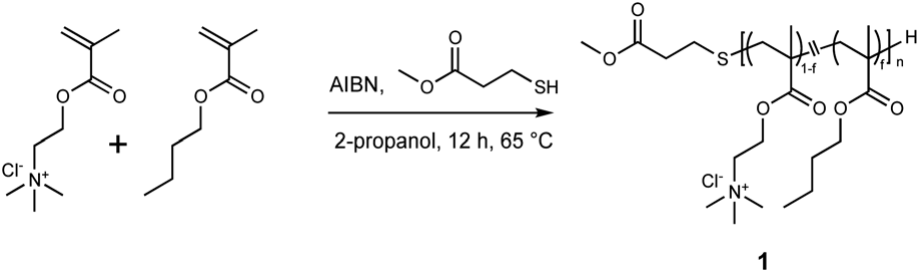
Synthetic route of nanodisc-forming polymer.

A nanodisc-forming amphiphilic polymethacrylate derivative was synthesized by free radical polymerization (Scheme 1). Methacroylcholine chloride (5.86 mL, 25 mmol), AIBN (82.1 mg, 500 μmol, 1 mol% of total monomers), methyl 3-mercaptopropionate (300 mg, 2.5 mmol, 20% of total monomers), and butyl methacrylate (3.97 mL, 25 mmol) were dissolved in 2-propanol (45 mL) in a round-bottom flask. The reaction mixture was bubbled with nitrogen for 5 minutes to remove the dissolved oxygen. The polymerization was performed by stirring the solution at 65 °C overnight. After the polymerization, the solvent was removed under vacuum. The crude polymer was dissolved in 1 mL of methanol and added to ice-cold diethyl ether. The precipitate was collected by centrifugation and lyophilized from an aqueous solution to give a cationic copolymer as a white powder (8.48 g, 92%). ^1^H NMR measurement was performed to determine the degree of polymerization (DP) and the mole fraction of butyl methacrylate (f) of polymers. The overlapped peak around 4.0 ppm assigned to CH̲_2_N(CH_3_)_3_^+^ for the cationic unit and C(=O)OCH̲_2_ for the hydrophobic unit was used to determine the number of polymerization. The integrations of methylene peak for C(=O)OCH̲_2_ on the cationic monomer unit (4.5 ppm, 2H) was used to determine the average number of the cationic unit. (Detailed calculation is shown in ESI†). ^1^H NMR (600 MHz, methanol-d_4_, TMS) for the polymer: *δ* 4.5 (m, 32.5H), 4.1–3.8 (m, 64.2H), 3.7 (s, 3H), 3.5–3.3 (m, 138H), 2.9–2.5 (m, 7H), 2.2–1.8 (m, 63.3H), 1.8–1.6 (m, 31.9H), 1.6–1.4 (m, 32.6H), 1.3–0.9 (m, 140H). ^1^H NMR spectrum is given in the ESI (Fig. S1†).

### Preparation of liposomes and nanodiscs

Liposomes were prepared by the hydration of lipid thin film. 147 μL of DPPC stock in methanol (10 mg mL^−1^) was evaporated under a stream of nitrogen gas. 1 mol% of Rh-DHPE was added to the lipid solution if needed. The obtained film was kept under vacuum for 3–5 h to completely remove the residual trace solvent. The resultant lipid film was hydrated by mixing with 1 mL PBS buffer (pH = 7.4, [NaCl] = 150 mmol L^−1^) at 55 °C and vortexed for 10 minutes. The obtained liposome dispersion was subjected to five cycles of freeze-and-thaw at −196 and 55 °C, followed by extrusion (11 times) through stacked 100 nm polycarbonate membrane filters installed in the LiposoFast mini extruder from Avestin. To form lipid nanodiscs, an appropriate amount of the polymer in PBS buffer (pH = 7.4, [NaCl] = 150 mmol L^−1^) was added to the dispersion of liposomes. The obtained liposome–polymer mixture was incubated using a water bath at 55 °C for 45 minutes. The concentration of the lipid was quantified by an enzymatic assay using choline oxidase and *N*-ethyl-*N*-(2-hydroxy-3-sulfopropyl)-3,5-dimethoxyaniline sodium salt.^24^

### Characterisation of nanodiscs

Prepared lipid nanodiscs were characterized by dynamic light scattering (DLS), differential scanning calorimetry (DSC), and transmission electron microscopy (TEM). The hydrodynamic diameter (*D*_hy_) of the lipid–polymer complexes was evaluated using a size analyser (ELSZ-1000ZS, Otsuka Electronics Co., Ltd., Osaka, Japan). Size distribution of the aqueous sample was obtained by analysing a time course of scattered light intensity at an angle of 165° from the incident light with the Contin method. The sample temperature was maintained at 25 °C using a thermostat temperature controller. DSC measurement of the nanodisc sample was carried out using a VP-DSC calorimeter (MicroCal, USA). The measurement was performed in the temperature range of 10 to 50 °C with a scanning rate of 0.8 °C min^−1^.

The specimen of liposomes and nanodiscs for TEM observation was prepared by the negative-stain and cryogenic method. An aliquot (2 μL) of sample solution was placed on a grid mesh (Cu 200, JEOL, 780111621) with a support film that had been subjected to a hydrophilic treatment for 1 minute using a glow discharger. After 1 minute of deposition, excess solution on the grid was blotted from the backside of the grid using filter paper. Then, the residual salt on the grid was washed with 2 μL of Milli-Q water twice. 2 μL of 2% aqueous solution of phosphotungstic acid was subsequently placed on a grid and left for 1 minute. An excess amount of phosphotungstic acid solution was removed using filter paper. Finally, the specimen was dried gently in a desiccator containing silica gel. For the cryogenic TEM (cryo-TEM) observation, the specimen was prepared using an EM GP2 automatic plunge freezer (Leica, Germany). The TEM observation was performed using JEM-2200FS (JEOL Ltd., Tokyo, Japan) at an acceleration voltage of 200 kV. The microscopic image was recorded using a Rio 9 CCD camera (Gatan, USA) installed in the microscope.

### Cell culture and viability test

HeLa cells were cultured at 37 °C in a humidified atmosphere with 5% CO_2_ in MEM which was enriched with 1% non-essential amino acid (NEEA), 1% penicillin–streptomycin, and 10% fetal bovine serum (FBS). The cytotoxicity of nanodisc and polymer was evaluated using a Cell Counting Kit-8 (CCK-8).^25^ Nanodiscs were prepared at various [DPPC]/[polymer] ratios ranging from 4 to 64. The prepared solution of nanodiscs or a nanodisc-forming polymer was serially diluted two-fold to set the final concentrations of [polymer] = 1.1–140 μmol L^−1^. HeLa cells were seeded at a density of 10 ×10^4^ cells per mL with 100 μL of the medium in a 96-well culture plate (Costar 3595, Corning). After overnight incubation, the medium was removed and the cells were washed with 100 μL of PBS (pH = 7.4, [NaCl] = 150 mmol L^−1^). Then, 90 μL of fresh medium and 10 μL of nanodisc or polymer sample were added to each well. After the incubation of the cells overnight or 48 hours, the medium was replaced and 10 μL of CCK-8 solution was added. Prior to the measurement, the plate was incubated at 37 °C with 5% CO_2_ for 3 hours. The absorbance at 450 nm was measured using a SpectraMax M5 plate reader (Molecular Devices, San Jose, CA, USA). The background absorbance was measured using a medium without cells and subtracted. PBS buffer (pH = 7.4, [NaCl] = 150 mmol L^−1^) was used as a positive control instead of nanodisc or polymer in the culture medium.

### Quantification of the cellular internalisation

Internalisation of nanodiscs and liposomes was quantified by fluorescence measurement using Rh-DHPE-labelled lipid nanodiscs. HeLa cells were plated at a density of approximately 20 × 10^5^ cells per mL with 1 mL of culture medium in 12-well plates (Costar 3513, Corning). A 10 mM stock solution of lipid nanodiscs and liposomes with 1% Rh-DHPE was prepared and diluted with PBS to the desired concentration. The final concentration of lipid (DPPC) was set to 1.56 μmol L^−1^ in the culture medium with HeLa cells. PBS was used as a negative control. After the exposure of HeLa cells to lipid nanodiscs or liposomes for 1 hour at 37 °C in 5% CO_2_ atmosphere, the cells were washed 3 times with 1 mL of PBS to remove excess nanodiscs or liposomes. To obtain the suspension of cells, the adhered HeLa cells were trypsinized. Collected cells were centrifuged at 1500 rpm for 3 minutes and washed twice with PBS at 37 °C. Finally, the cells were resuspended in 1 mL PBS and the number of cells was counted using a Neubauer-improved cell counting chamber. The fluorescence intensity of cellular suspension was measured using a JASCO FP-8300 fluorescence spectrometer (Tokyo, Japan). Emission intensity at 585 nm was recorded with the excitation at 560 nm. Excitation and emission band-passes were set to 10 nm. The fluorescence intensity was normalised against 10^4^ cells.

### Fluorescence microscopy of HeLa cells

Localization of nanodisc or liposome in HeLa cells was observed by a fluorescence microscope. For the fluorescence imaging, the liposome and lipid nanodiscs were stained with 1 mol% of Rh-DHPE. Cultured HeLa cells in the exponential growth phase were washed, trypsinized, and resuspended in the culture medium. 2 mL of culture medium containing approximately 10^5^ cells per mL was placed in a glass bottom dish coated with poly-l-lysine (D11131H, Matsunami). The dishes were incubated at 37 °C in a 5% CO_2_ atmosphere overnight to complete the adhesion of cells to the glass surface of the dish. The growth medium was aspirated from each dish, and the adhered HeLa cells were washed with 1 mL PBS. After the addition of 2 mL of the culture medium, 20 μL of nanodisc or liposome samples were added to the dishes and the cells were further incubated at 37 °C in a 5% CO_2_ atmosphere for the desired time. After the incubation, the medium and untrapped nanodiscs or liposomes were removed by aspiration. Prior to the microscopic observation, the dish was washed with PBS. Microscopic observation was carried out using an Olympus IX71 inverted fluorescence microscope (Tokyo, Japan) equipped with an Olympus LUM Plan FLN 20× objective lens (Tokyo, Japan) and a U-MWIG2 filter unit (excitation 520–550 nm, emission > 580 nm). Fluorescence images were acquired using a Hamamatsu ORCA-spark CCD camera (Hamamatsu, Japan). Confocal microscopy was conducted using an Olympus IX83 inverted fluorescence microscope (Tokyo, Japan) equipped with a Hamamatsu MAICO® MEMS confocal unit (Hamamatsu, Japan). For the colocalisation study, cell nuclei were stained with a NucleoSeeing® fluorescent probe.^26^

### Preparation of PTX-loaded nanodiscs and their anticancer activity

PTX-loaded liposomes and nanodiscs were prepared by the film-hydration method as described above. 3 mol% of PTX dissolved in methanol was further added for the preparation of lipid thin films. Obtained samples of liposome and lipid nanodisc were centrifuged at 4500 rpm for 45 minutes to remove the unentrapped drug. 10 μL of supernatant was diluted with 990 μL methanol to solubilize lipid aggregates, which show a strong background in the absorption spectrum. The molar concentration of PTX was measured by a Hitachi U-3310 spectrophotometer (Tokyo, Japan) using a molar extinction coefficient of 29 800 M^−1^ cm^−1^ at 227 nm.^27^ The final loading efficiency of PTX was calculated as follows:Loading efficiency (%) = (loaded amount of PTX determined by absorption)/(amount of PTX employed for preparation) × 100

The loading efficiency of PTX was estimated by averaging the results three times. The drug release study was performed by dialysis using a Pur-A-Lyzer™ Midi 1000. 800 μL of the nanodiscs with a [DPPC]/[polymer] ratio of 8 was introduced to the dialysis tube. The concentrations of DPPC and PTX were set to 3.9 mmol L^−1^ and 118 μmol L^−1^, respectively. The nanodisc sample was dialysed in 1 L of PBS buffer and the amounts of residual PTX at 0, 1, 3, 6, 12, 24, 36 and 48 hours were quantified using a NanoPhotometer N60 (Implen, Germany).

DPPC liposomes and lipid nanodiscs with a [DPPC]/[polymer] ratio of 4, 8, 16, 32, and 64 were prepared. For all samples, the final concentrations of DPPC and PTX were set to 3.12 μmol L^−1^ and 94 nmol L^−1^, respectively. The anti-cancer activity of PTX-loaded liposomes and nanodiscs was assessed by a CCK-8 assay as described above. HeLa cells were exposed to PTX-loaded liposomes or nanodiscs for 48 hours at 37 °C in a 5% CO_2_ atmosphere prior to the viability measurement. PBS (pH = 7.4, [NaCl] = 150 mmol L^−1^) was used as a positive control instead of samples in the culture medium. Results were expressed as mean ± standard deviation of three independent experiments.

## 3 Results and discussion

### Preparation and characterisation of lipid nanodiscs

**Fig. 1:**
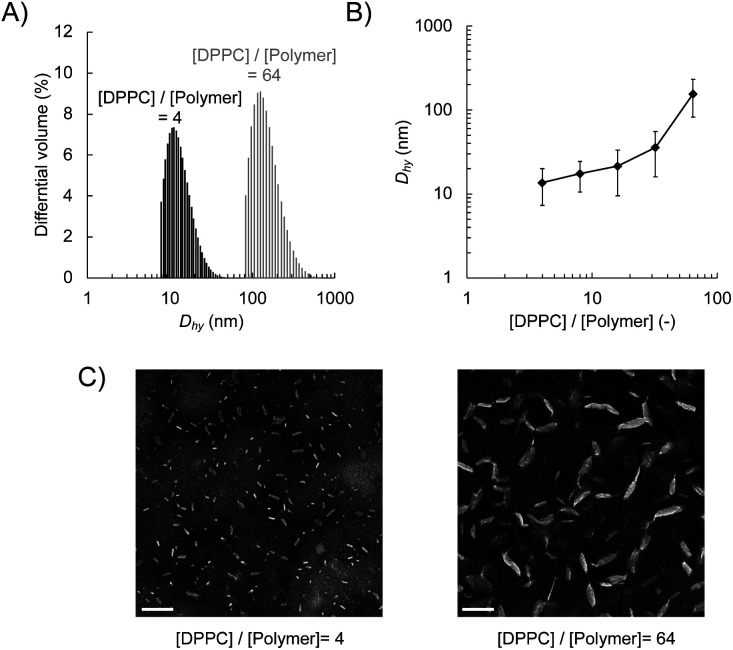
Formation of the discoidal polymer–lipid complex by DPPC and an amphiphilic polymethacrylate derivative. (A) Size distribution of polymer–lipid complex and (B) the effect of [DPPC]/[polymer] ratio on the *D*_hy_ evaluated by DLS. Error bars represent the polydispersity. (C) Negative-stain TEM images of lipid nanodiscs prepared at [DPPC]/[polymer] = 4 and 64. Scale bar = 100 nm, [DPPC] = 100 μmol L^−1^, in PBS buffer (pH = 7.4, [NaCl] = 150 mmol L^−1^) evaluated at 25 °C.

We prepared lipid nanodiscs according to the procedure described previously.^20^ An amphiphilic polymethacrylate random copolymer with cationic and hydrophobic side chains was synthesised by a free radical copolymerisation of butyl methacrylate and methacryloyl choline chloride in the presence of methyl 3-mercaptopropionate as a chain transfer agent (Scheme 1). The resulting polymer was characterised by ^1^H NMR. The fraction of hydrophobic unit (*f*), the degree of polymerisation (DP), and the corresponding number-averaged molecular weight (*M*_n_) were found to be 0.49, 32, and 5750 g mol^−1^, respectively. The lipid–polymer complex was prepared by mixing the polymer with large unilamellar vesicles (LUVs) composed of DPPC at 55 °C, which is above the gel-to-liquid crystalline phase transition temperature of DPPC (41 °C). We evaluated the hydrodynamic diameter (*D*_hy_) of obtained lipid–polymer complex by DLS (Fig. 1A). The average *D*_hy_ of the lipid–polymer complex prepared with [DPPC]/[polymer] ratio of 4 and 64 were found to be 13 nm (PDI = 0.087) and 216 nm (PDI = 0.210), respectively. The [DPPC]/[polymer] ratio dependence of *D*_hy_ suggested that as the amount of polymer relative to lipid increases, smaller lipid–polymer complexes are formed (Fig. 1B). In this study, we used DPPC as the lipid component for nanodisc formation that is different from our previous report using 1,2-dimyristoyl-*sn*-glycero-3-phosphocholine (DMPC).^20^ Temperature-dependent DLS measurement of the sample prepared with [lipid]/[polymer] ratio of 8 revealed the improved thermal stability of DPPC-based nanodiscs. In particular, DPPC-based nanodiscs showed no significant size variation between 15 °C and 37 °C, whereas a significant increase in nanodisc size was observed at 37 °C for the DMPC-based system (Fig. S2†). The presence of lipid bilayer in nanodiscs was verified through phase transition measurement using DSC. The DSC thermogram for the sample prepared with [DPPC]/[polymer] ratio of 8 exhibited endothermic peaks at 41 °C with a shoulder at 35 °C (Fig. S3†), corresponding to the main transition from lamellar rippled gel (P_β′_) to liquid crystalline (L_α_) phase and the pre-transition from lamellar gel (L_β′_) to P_β′_ phase, respectively.^28^ This observation suggested that the encompassed lipid bilayer maintained the lamellar structure.

Negative-stain TEM confirmed the formation of discoidal nanoparticles (Fig. 1C). The image analysis revealed that the diameter of the nanodisc with the [DPPC]/[polymer] ratio of 4 was found to be 18.3 nm ± 8.4 nm, which is significantly smaller than that with a ratio of 64 (76.1 nm ± 32.6 nm). Taken together with the results of DLS measurements, it was demonstrated that lipid nanodiscs with various sizes can be prepared by changing the [DPPC]/[polymer] ratio. Furthermore, cryo-TEM observation did not find any large lipid aggregates such as liposomes in the prepared nanodisc sample, thereby confirming their homogeneity (Fig. S4B†). From the images of the edge-on nanodiscs, their thickness was found to be 5 nm, which corresponds to the thickness of a single lipid bilayer (Fig. S4B†). This observation further supports the presence of a lipid bilayer structure within the nanodisc.

### Interaction of lipid nanodiscs and intact HeLa cells

We investigated the interaction of the lipid nanodisc with intact cells. The *in vitro* cytotoxicity caused by the exposure of the lipid nanodisc and nanodisc-forming polymer to HeLa cells was evaluated by a CCK-8 assay.^25^ The 50% lethal concentrations (LC_50_) for the polymer alone and the nanodisc were measured after the exposure overnight or for 48 hours (Table 1 and Fig. S5†). For overnight incubation, the LC_50_ values of the nanodisc and polymer alone were found to be in a range of 5.3 to 6.5 μmol L^−1^, indicating that complexation of lipid does not affect the toxicity of the polymer itself. Prolonged exposure for 48 hours resulted in a slight decrease in the LC_50_ values for all cases. All the experiments employing intact cells in this study were performed at sufficiently lower polymer concentrations than the LC_50_ where the cytotoxicity of nanodisc is negligible. We quantified the cellular uptake of the nanodisc by fluorescence measurement. The nanodisc was prepared with a [DPPC]/[polymer] ratio of 8 and labelled with 1 mol% of Rh-DHPE. After the exposure of Rh-DHPE labelled nanodiscs for 1 hour at 37 °C, HeLa cells adhered to a dish were washed with PBS buffer (pH = 7.4) to remove excess nanodiscs that were not incorporated into cells. We measured the rhodamine fluorescence of the cellular dispersion obtained by trypsinisation of adhered HeLa cells to quantify the incorporated nanodiscs. The fluorescence intensity normalised to a constant number of cells gradually increased with the concentration of nanodiscs (Fig. S6†). These results clearly indicated that the cellular uptake of lipid nanodisc is induced at sufficiently low concentrations that do not show cytotoxicity.

**Table 1:** Cytotoxicity of polymer and lipid nanodiscs determined by CCK-8 assay

	[DPPC]/[polymer] (—)	LC_50_ ([polymer]/μmol L^−1^)	
		Overnight	48 hours
Polymer	—	6.0	5.0
Nanodisc	4	6.0	4.9
	8	6.0	4.5
	16	6.2	4.7
	32	6.5	4.7
	64	5.3	3.7

**Fig. 2:**
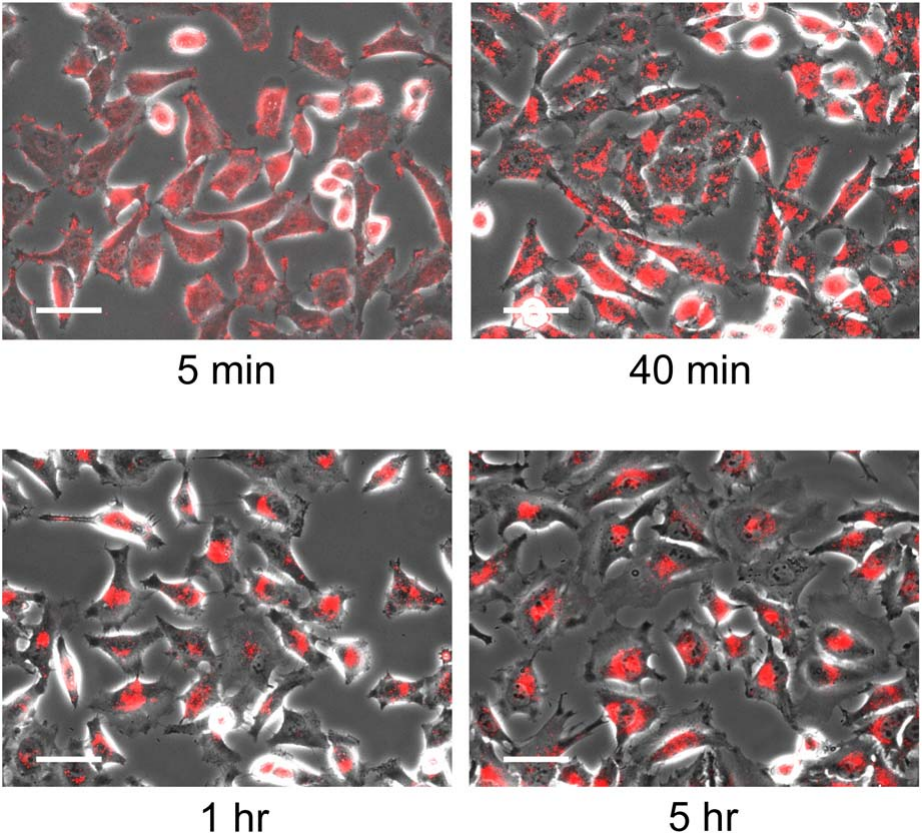
Time-dependent uptake of nanodiscs by HeLa cells. The cells were exposed to nanodiscs at 37 °C for the indicated times (5 minutes to 5 hours). Phase contrast image was overlayed with fluorescence image. [DPPC]/[polymer] = 8, [DPPC] = 1.75 μmol L^−1^, scale bar = 50 μm.

The localisation of the nanodisc in HeLa cells was evaluated in detail by microscopic observation. As a control experiment, we confirmed that microscopic observation of HeLa cells in the absence of nanodiscs as well as the addition of unlabelled nanodiscs showed no fluorescence signal, indicating that the fluorescence signal corresponds to Rh-DHPE labelled on nanodiscs (Fig. S7A and B†). Time-lapse observation of the HeLa cells in the presence of nanodiscs with a [DPPC]/[polymer] ratio of 8 revealed that the incubation time of the nanodiscs and cells significantly affects the subcellular localisation of the nanodiscs (Fig. 2). At an early stage (5 minutes), fluorescent signals were homogeneously observed over the cells, reflecting that the nanodiscs were bound to cells without specific localisation. By prolonged incubation, fluorescent signals were found to be localised to a specific location; the nanodiscs were localised into the cytoplasm in 40 minutes and then delivered to the vicinity of the cell nucleus (1 hour). Since further incubation for 5 hours did not show any significant change in the localisation of the fluorescent signal, we found that the nanodiscs incorporated into cells are finally delivered to the periphery of the nucleus. We did not observe any fluorescence signal inside the cells when Rh-DHPE alone was added without nanodiscs (Fig. S7C†), suggesting that the observed accumulation of fluorescence signal was due to the Rh-DHPE delivered by incorporated nanodiscs. To examine detailed localisation, we further performed confocal microscope observation (Fig. S8†). We observed that the Rh-DHPE signal corresponding to nanodisc was widely distributed in a cell after 5 minutes of exposure, indicating the incorporation of nanodiscs to the cytoplasm. Prolonged incubation for 1 hour resulted in the localisation of Rh-DHPE signal close to the nucleus and some signals overlapped with the nucleus.

**Fig. 3:**
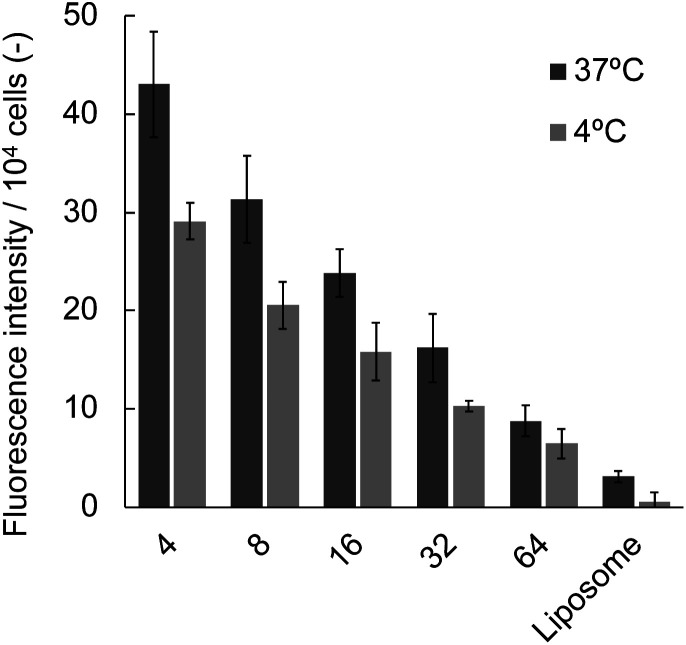
Effect of [DPPC]/[polymer] ratio on the internalisation of nanodisc into HeLa cells determined by fluorescence measurement. [DPPC] = 1.56 μmol L^−1^, HeLa cells were exposed to nanodiscs or liposomes at 37 °C and 4 °C for 1 hour.

**Fig. 4:**
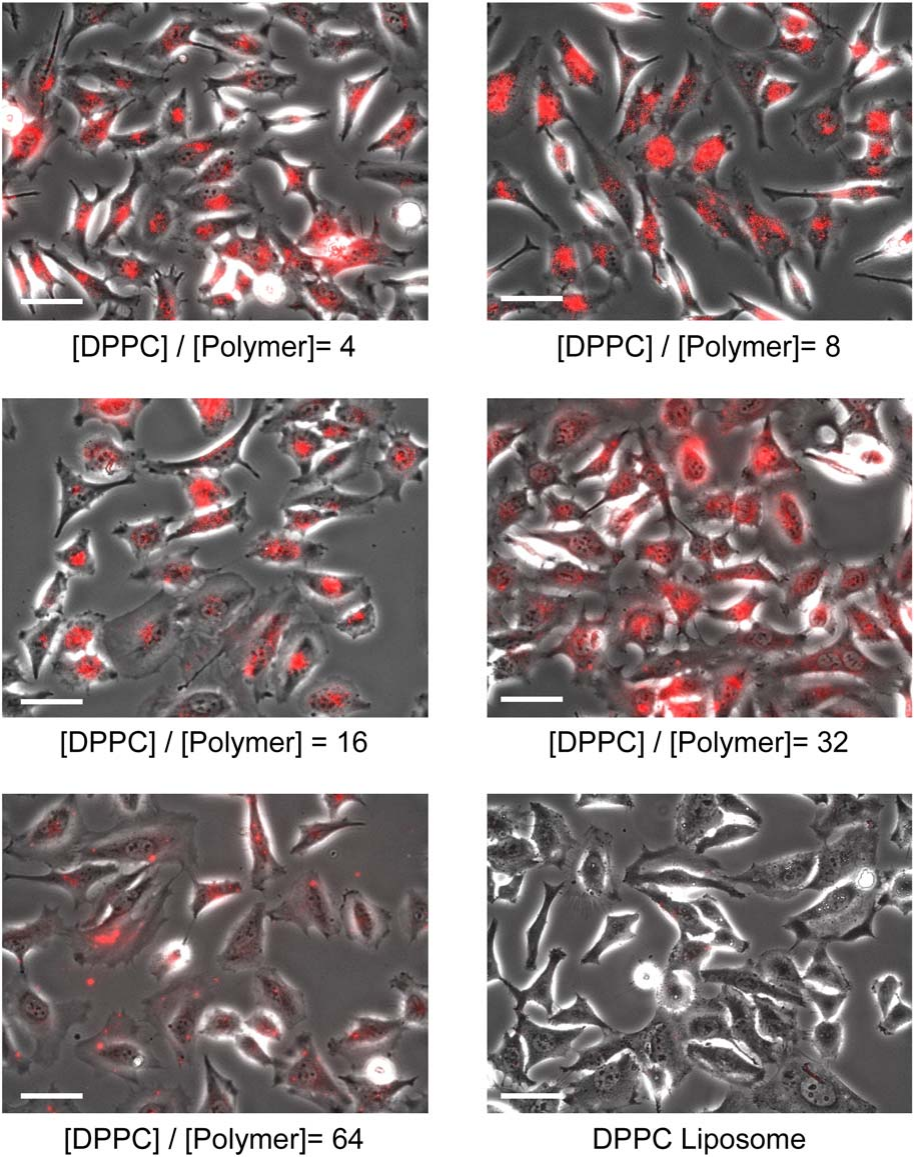
Microscopic observation of the nanodisc localisation inside HeLa cells. [DPPC] = 1.75 μmol L^−1^, cells were incubated in the presence of nanodiscs or liposomes at 37 °C for 1 hour, scale bar = 50 μm.

We also examined the influence of the [DPPC]/[polymer] ratio employed for the nanodisc formation on cellular uptake by fluorescence measurement. Nanodisc samples were prepared at a constant DPPC concentration (1.56 μmol L^−1^) with various amounts of the polymer. The uptake of nanodiscs was found to be significantly enhanced by a decreasing [DPPC]/[polymer] ratio at both incubation temperatures (4 and 37 °C, Fig. 3). Noteworthy, all the nanodisc samples showed significantly higher fluorescence than the liposome, reflecting their high efficiency of cellular internalisation. Taken together with the fact that the increase of the polymer fraction resulted in the formation of smaller nanodiscs as described above (Fig. 1), it is likely that the smaller nanodiscs are incorporated into the cells more efficiently compared to larger ones. In addition, an increase in the total amount of cationic polymers that strongly interact with the negatively charged surfaces of HeLa cells may promote the internalisation of the nanodiscs. Since the addition of free polymer to the nanodisc sample did not affect the amount of internalised nanodiscs (Fig. S9†), it is evident that the coexistence of the non-bound polymer in the system does not contribute to cellular uptake. The uptake of liposomes was not observed at 4 °C, where the endocytosis-mediated pathway of internalisation is inhibited.^29^ In contrast, the uptake of the nanodisc was induced even at the same temperature, suggesting that the uptake of nanodiscs is driven by an energy-independent passive pathway. Cellular uptake of both nanodiscs and liposomes was further enhanced at 37 °C where the energy-requiring endocytosis is active, suggesting that nanodisc uptake at physiological temperatures is simultaneously governed by passive and active transport pathways. Microscopic observation was further performed to identify the localisation of nanodiscs depending on the [DPPC]/[polymer] ratio (Fig. 4). We did not observe significant fluorescence signals inside the cell for the liposome, reflecting that the uptake was hardly induced. Nanodisc systems showed the localisation of the fluorescence signal varied depending on the [DPPC]/[polymer] ratio. For the nanodisc prepared at the [DPPC]/[polymer] ratios of 32 and 64, the fluorescence signal was distributed to the cytoplasm; whereas, nanodiscs prepared at the [DPPC]/[polymer] ratio of 16 and smaller were found to be localised to the vicinity of the cell nucleus. Confocal microscopy confirmed that the nanodisc prepared at the [DPPC]/[polymer] ratio of 8 was delivered to the vicinity of the nucleus (Fig. S8B†). On the other hand, for the nanodisc formed with the [DPPC]/[polymer] ratio of 32, the Rh-DHPE signals were widely distributed in a cytoplasm without showing specific overlap with the nucleus (Fig. S8C†). Although the detailed mechanism of the uptake pathway is beyond the scope of this paper, the geometry of nanodiscs including their discoidal shape and size was found to influence the efficiency of cell internalisation.^30^

### Lipid nanodisc as a nanocarrier for anticancer agent

We have investigated the potential of the lipid nanodisc as a carrier for the delivery of anticancer drugs. We employed PTX, which expresses its anticancer activity by the suppression of microtubule dynamics.^31^ Since PTX displays poor solubility in water due to its hydrophobic nature, its administration requires molecular carriers with a hydrophobic interior. 3 mol% of PTX was loaded to DPPC liposomes prior to nanodisc formation. The loading efficiency was estimated by the absorption of PTX at *λ* = 227 nm (Fig. S10†) and found to be 89% (Table 2). The addition of the polymer to PTX-loaded liposomes did not show a significant decrease in the loading efficiency (88–89%), reflecting that the nanodiscs maintain a hydrophobic interior of the lipid bilayer for the entrapment of PTX. DLS measurement revealed that the diameter of the nanodisc slightly increased with the incorporation of PTX (Table 2). Negative-stain TEM observation was performed to observe PTX-incorporated liposomes and nanodiscs (Fig. S11†). The marked difference in the morphology of lipid assembly was not observed for either system, except that a small fraction of nanodiscs were aggregated in the presence of PTX. The size distribution of the nanodiscs obtained by the image analysis also confirmed a slight increase in the diameter (Fig. S12†). Time-course of the PTX release from the nanodiscs was evaluated by a dialysis method (Fig. S13†).^32^ We found that the PTX incorporated in the nanodiscs was gradually released into the aqueous phase. The accumulated release of PTX from the nanodisc for 48 hours was found to be approximately 40% (37 nmol L^−1^), indicating that the majority of PTX molecules were retained in the nanodisc probably due to its low solubility in water.

**Table 2:** Characterisation of DPPC liposome and lipid nanodiscs in the absence and presence of 3 mol% of PTX. [DPPC] = 1 mmol L^−1^, [DPPC]/[polymer] = 4, 8, 16 for nanodisc samples, [PTX] = 30 μmol L^−1^, in PBS (pH = 7.4, [NaCl] = 150 mmol L^−1^) measured at 25 °C

	[DPPC]/[polymer] (—)	*D*_hy_ (nm)	Loading efficiency (%)
Liposome	—	105	N/A
Nanodisc	4	13.4	N/A
	8	15.7	N/A
	16	21.0	N/A
3 mol% PTX-loaded liposome	—	114	88.6 ± 1.2
3 mol% PTX-loaded nanodisc	4	15.0	87.7 ± 0.3
	8	17.1	88.3 ± 0.3
	16	23.8	88.5 ± 0.5

**Fig. 5:**
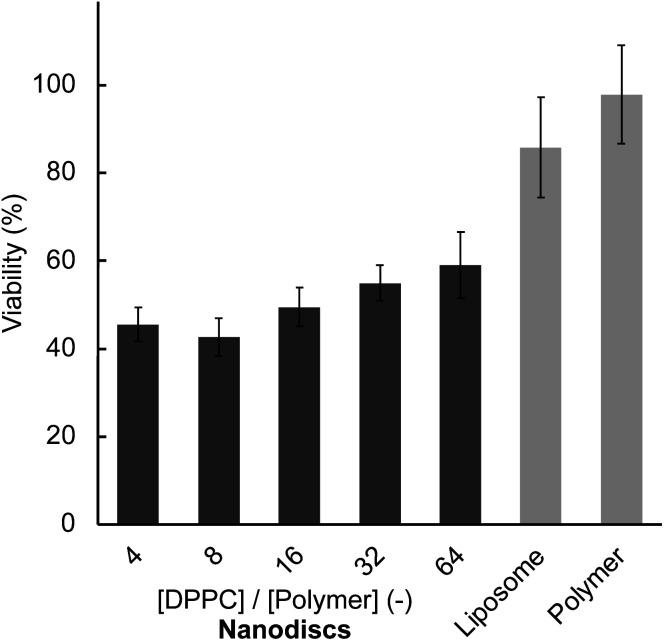
Anticancer activity of PTX loaded in lipid nanodiscs and liposome, [DPPC] = 3.12 μmol L^−1^, [PTX] = 94 nmol L^−1^, [DPPC]/[polymer] = 4–64, HeLa cells were incubated at 37 °C for 48 hours. The PTX activity for polymer alone was measured at [polymer] = 0.78 μmol L^−1^.

Finally, the anticancer activities of PTX-loaded liposomes and nanodiscs were examined by measuring the viability of HeLa cells after 48 hours of incubation. Prior to viability measurement, we confirmed that the general trend in the cellular uptake at 48 hours did not change from the short time (1 hour) incubation (Fig. S14†). All nanodisc samples displayed significantly lower cell viability (40 to 60%) compared to liposome (85%) at a constant amount of lipid and PTX (Fig. 5), reflecting the enhanced anticancer activity. Also, the polymer–PTX mixture in the absence of lipids did not show anticancer activity (98% viability), suggesting that the presence of a lipid membrane is essential for the delivery of PTX. The concentration of nanodisc employed for anticancer activity measurement (0.78 μmol L^−1^ in maximum) was significantly lower than the LC_50_ values after 48 hours of incubation (Table 1), indicating that observed cancer-killing activity originated in PTX. This enhanced anticancer activity of the nanodiscs is likely due to their excellent cellular uptake efficiency compared to liposomes as described above.

## 4 Conclusions

We have investigated the interaction of lipid nanodiscs and intact HeLa cells to demonstrate their potential for molecular delivery. The lipid nanodiscs were prepared simply by the spontaneous fragmentation of liposomal membrane and their size can be tuned by changing the [DPPC]/[polymer] ratio. The lipid nanodisc displayed excellent uptake efficiency compared to liposomes at a concentration where nanodiscs do not show cytotoxicity. The [DPPC]/[polymer] ratio significantly affects the internalisation efficiency of nanodiscs as well as their localisation inside the cells. The nanodisc encapsulating PTX displayed significantly higher anticancer activity than PTX-loaded liposomes against HeLa cells, reflecting their excellent potential to deliver payloads to intact cells. The properties of polymer-based lipid nanodiscs can be easily modulated by varying both the lipid composition and the chemical structure of the polymers, allowing for precise fine-tuning of their biological functionality. The lipid nanodisc technology demonstrated in this study is expected to provide a new approach to designing an excellent nanocarrier for drug delivery applications.

## Author contributions

J. Hao: formal analysis, investigation, methodology, visualization, writing – original draft. M. Ishihara: investigation, methodology. G. Rapenne: resources, supervision, writing – review & editing. K. Yasuhara: conceptualization, data curation, formal analysis, funding acquisition, investigation, methodology, project administration, resources, supervision, validation, visualization, writing – original draft, writing – review & editing.

## Conflicts of interest

There are no conflicts to declare.

## Supplementary Material

RA-014-D3RA07481A-s001
