# Supplementary material for: Lipid nanodiscs spontaneously formed by an amphiphilic polymethacrylate derivative as an efficient nanocarrier for molecular delivery to intact cells
Source: RSC Adv. 2024 Feb 19;14(9):6127–34. doi: 10.1039/d3ra07481a (PMC10875731; doi:10.1039/d3ra07481a)
Supplement: RA-014-D3RA07481A-s001 [file RA-014-D3RA07481A-s001.pdf]

Electronic Supplementary Material (ESI) for RSC Advances.
This journal is © The Royal Society of Chemistry 2024

Supporting Information for

Lipid nanodiscs spontaneously formed by an amphiphilic polymethacrylate derivative as an efficient nanocarrier for molecular delivery to intact cells

Jinyu Hao[a], Mika Ishihara[a], Gwénaël Rapenne[a, b] and Kazuma Yasuhara[a, c*]

a. Division of Materials Science, Nara Institute of Science and Technology (NAIST), 8916-5 Takayama-cho, Ikoma, 630-0192, Japan.
b. CEMES-CNRS, Université de Toulouse, CNRS, 29 Rue Marvig, F-31055 Toulouse Cedex 4, France
c. Centre for Digital Green-innovation, Nara Institute of Science and Technology (NAIST), 8916-5 Takayama-cho, Ikoma, 630-0192, Japan

TABLE OF CONTENTS

1. Characterization of the nanodisc-forming polymer
Fig. S1. ^{1}H NMR spectrum of nanodisc-forming polymer
Calculation of the structural parameters

2. Additional Experimental data
Fig. S2. Effect of temperature on the size distribution of nanodiscs prepared with different lipid compositions measured by DLS
Fig. S3. DSC thermogram for nanodiscs
Fig. S4. Cryo-TEM image of nanodiscs
Fig. S5. Viability of HeLa cells upon exposure to lipid nanodisc
Fig. S6. Effect of nanodisc concentration on the uptake by HeLa cells
Fig. S7. Additional microscopic images of HeLa cells as a control experiment
Fig. S8. Confocal microscopic images of HeLa cells after the exposure to nanodisc
Fig. S9. Effect of free polymer on the uptake of nanodiscs
Fig. S10. Absorption spectrum of PTX
Fig. S11. TEM images of PTX-loaded liposomes and nanodiscs
Fig. S12. Size distribution of nanodiscs based on the TEM image analysis
Fig. S13. Paclitaxel release study by dialysis
Fig. S14. Internalization of nanodisc into HeLa cells after 48 hours of incubation

1. Characterization of the nanodisc-forming polymer

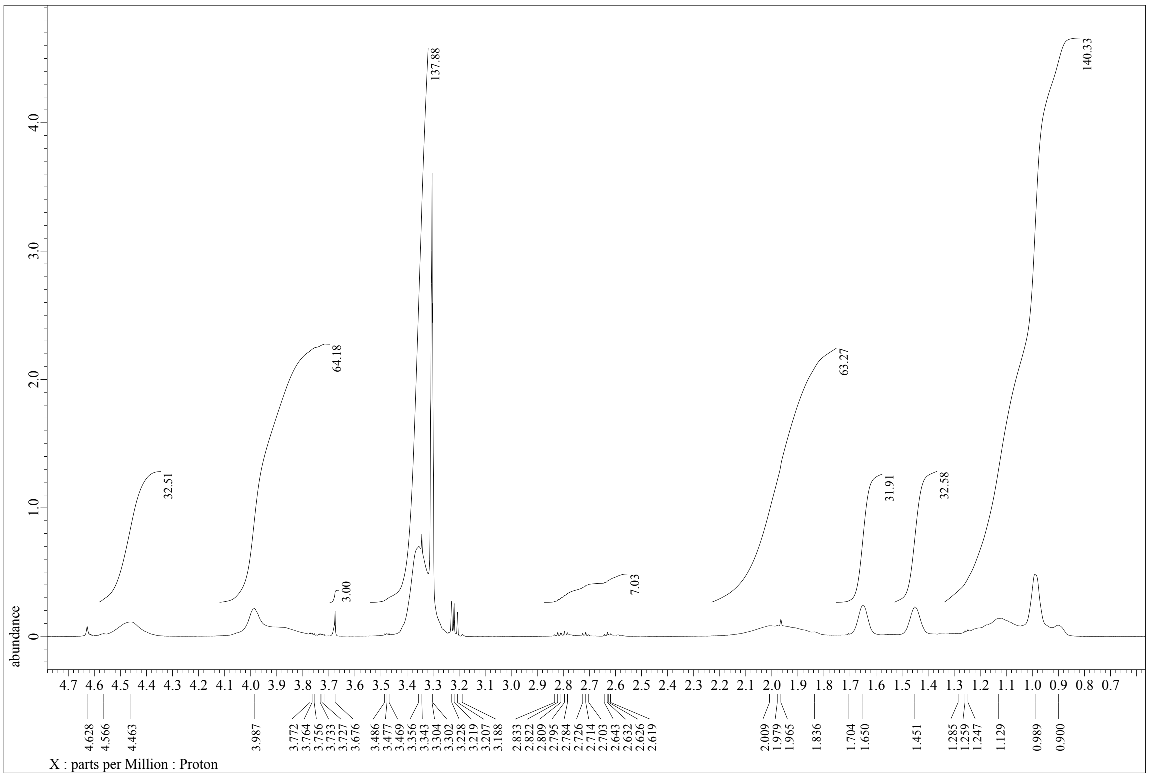

Fig. S1. ^{1}H NMR spectrum of nanodisc-forming polymer (600 MHz, methanol-d_4, 293 K).

Calculation of the structural parameters

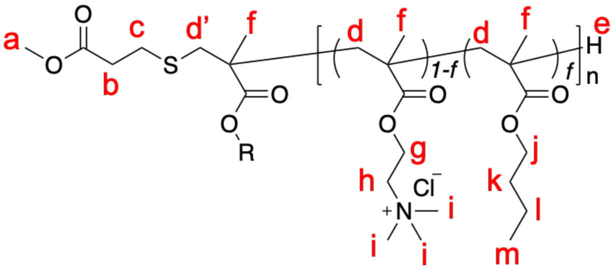

δ/ppm	Assignment	Interal ratio
0.9 - 1.3	f, m	140.33
1.4 -1.6	l	32.58
1.6 -1.8	k	31.91
1.8 - 2.2	d	63.27
2.5 - 2.9	b, c, d', e	7.03
3.3 - 3.5	i	137.88
3.7	a	3
3.7 - 4.1	h + j	64.18
4.4 - 4.6	g	32.51

The integral value of the methyl group proton (3.7 ppm, 3H, a) at the terminal of the main chain was normalised to 3. The specific calculation method is described below.

<u>The number of methacroylcholine chloride units per individual polymer chain (n_{cation})</u>

The integration of C(=O)O<u>CH$_2$</u> protons on the methacrylcholine chloride unit at 4.4 – 4.6 ppm (g) is 32.51 H. Therefore,

$32.51 = n_{cation} \times 2$

n_{cation} was found to be 16.26 (≒ 16).

<u>Degree of polymerization (DP)</u>

The methylene peak for <u>CH$_2$</u>N(CH$_3$)$_3^+$ on the methacrylcholine chloride unit (h) and the methylene peak for C(=O)O<u>CH$_2$</u> on the butyl methacrylate (j) overlapped at around 4.0 ppm and its integration is 64.18H. Therefore,

$64.18 = DP \times 2$

DP was found to be 32.09 (≒ 32).

<u>Fraction of hydrophobic unit (*f*)</u>

Since two different monomer units have been employed, *f* can be obtained by following the equation.

$f = 1 - n_{cation} / DP = 1 - 16.26 / 32.09 = 0.49$

The number-averaged molecular weight (M_n)

M_n is the sum of the molecular weight of the methacrylcholine chloride unit × the average number of methacrylcholine chloride units (n_{cation}), the molecular weight of the butyl methacrylate unit × the average number of butyl methacrylate units ($DP-n_{cation}$), and the molecular weight of the chain transfer agent.

$207.70 \times 16.26 + 142.20 \times (32.09 - 16.26) + 120.17 ≒ 5750$

Therefore, the M_n is found to be 5,750 g mol^{-1}.

2. Additional experimental data

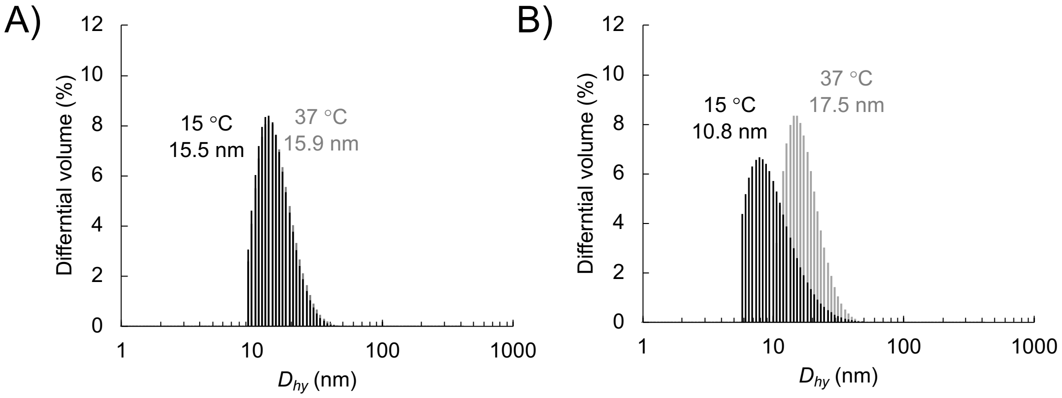

Fig. S2. Effect of temperature on the size distribution of nanodiscs prepared with (A) DPPC and (B) DMPC measured by DLS. [Lipid] / [Polymer]= 8, [lipid]= 1 mmol·L^{-1}, in PBS buffer (pH= 7.4, [NaCl]= 150 mmol·L^{-1})

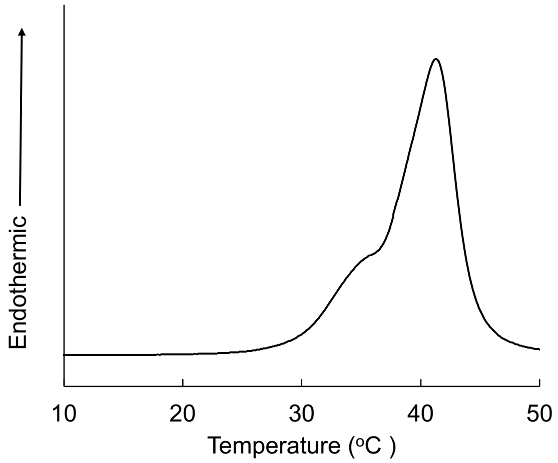

Fig. S3. DSC thermogram for DPPC nanodiscs. [DPPC] / [Polymer] = 8. [DPPC]= 10 mmol·L^{-1}, PBS buffer (pH= 7.4, [NaCl]= 150 mmol·L^{-1}). PBS buffer was used as a reference.

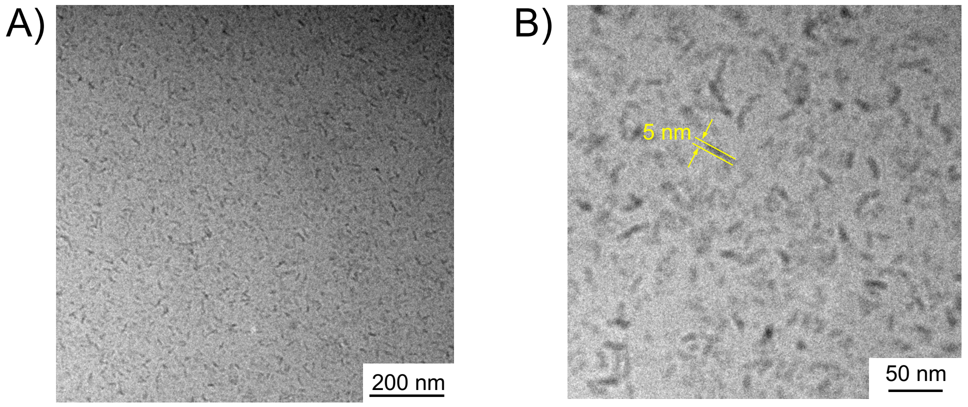

Fig. S4 Cryo-TEM images of nanodiscs. Panel (B) corresponds to the magnified image of the same sample as (A). [DPPC] / [Polymer] = 8. [DPPC]= 10 mmol·L^{-1}, PBS buffer (pH= 7.4, [NaCl]= 150 mmol·L^{-1}).

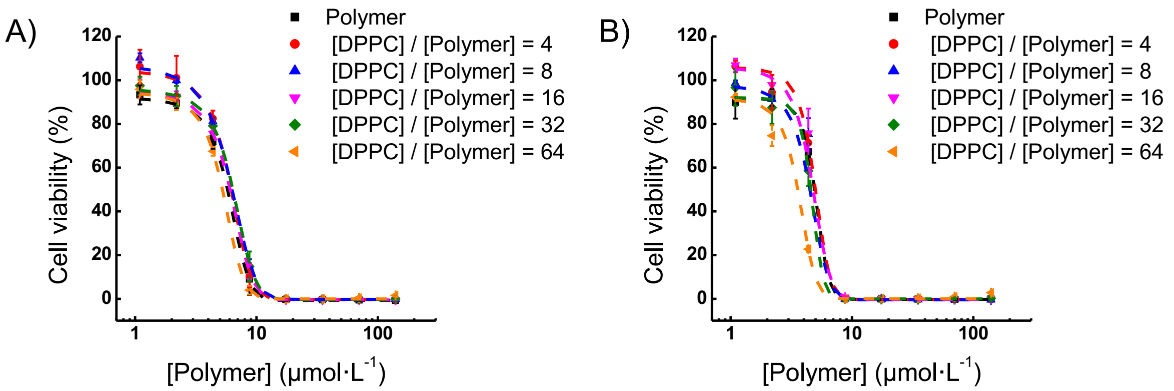

Fig. S5. Viability of HeLa cells upon exposure to lipid nanodisc and nanodisc-forming polymer. The measurement was performed after (A) overnight and (B) 48 hours of incubation at 37°C.

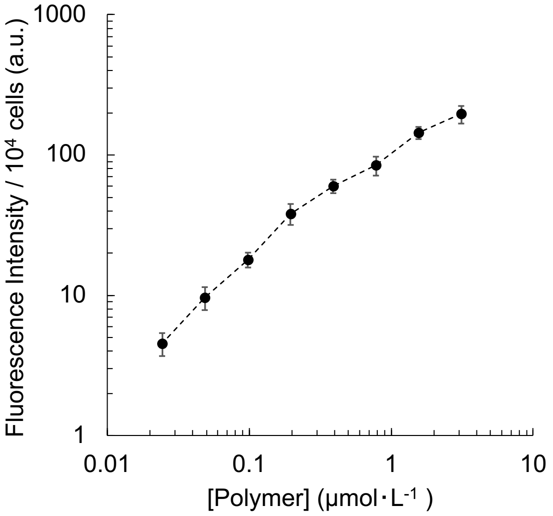

Fig. S6. Concentration dependence of the nanodisc internalisation by HeLa cells. [DPPC] / [Polymer] = 8, [DPPC] = 0.20-25 μmol·L^{-1}, incubated with HeLa cells at 37°C for 1 h.

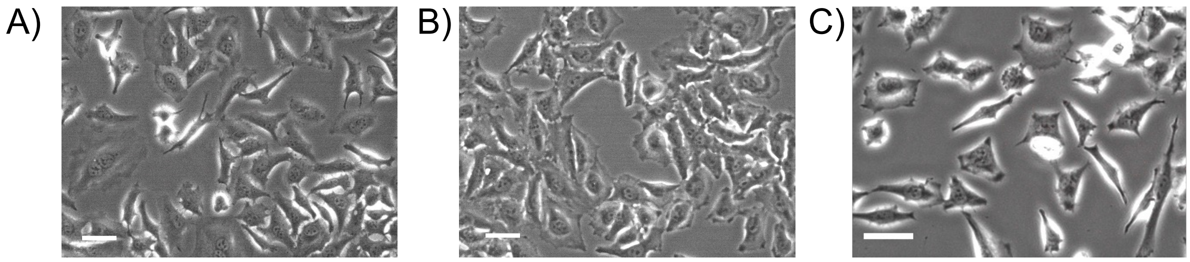

Fig. S7. Additional microscopic images of HeLa cells (A) in the absence of nanodisc, (B) in the presence of nanodiscs without Rh-DHPE and (C) in the presence of Rh-DHPE alone. The images (B) and (C) were acquired 1 hour after the sample addition at 37°C. Scale bar = 50 μm. [DPPC] = 1.75 μmol ·L^{-1}, [DPPC] / [polymer] = 8 for (B) and [Rh-DHPE] = 0.0175 μmol ·L^{-1} for (C).

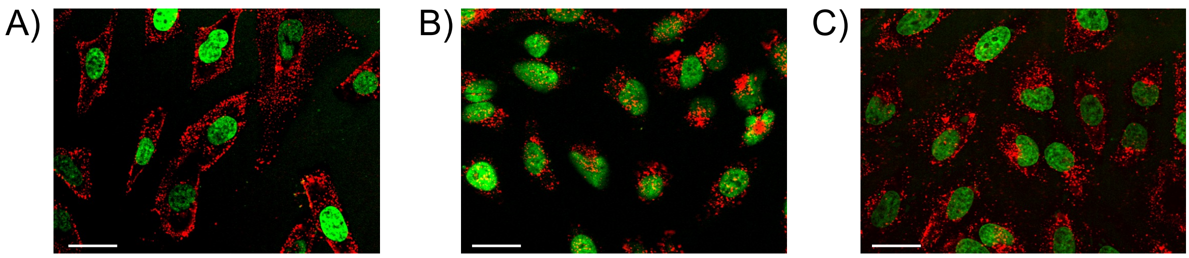

Fig. S8. Confocal microscopic images of HeLa cells (A) 5 minutes and (B) 1 hour after the addition of nanodiscs prepared at the [DPPC] / [Polymer] ratio of 8. Image (C) was acquired 1 hour after the addition of nanodiscs prepared at the [DPPC] / [Polymer] ratio of 32. cell nuclei and nanodiscs were stained with a NucleoSeeing® fluorescent probe (green) and Rh-DHPE (red), respectively. [DPPC]= 1.75 μmol·L^{-1}, scale bar= 30 μm.

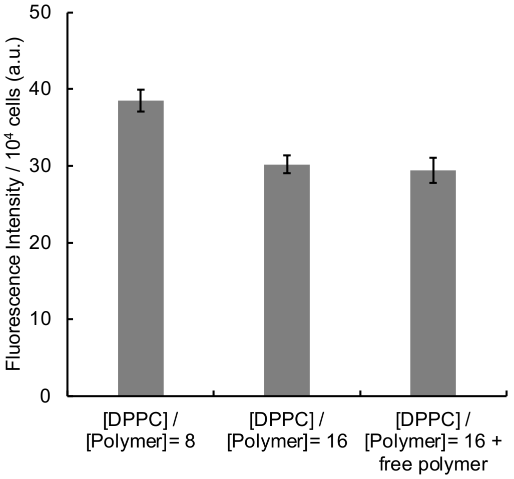

Fig. S9. Effect of free polymer on the uptake of nanodiscs. For the [DPPC]/[Polymer] = 16 + free polymer sample, an equal amount of polymer was added after nanodisc preparation, making the final polymer concentration the same as for the [DPPC]/[Polymer] = 8 sample. [DPPC] = 1.56 μmol·L^{-1}. HeLa cells were exposed to nanodiscs at 37 °C for 1 hour.

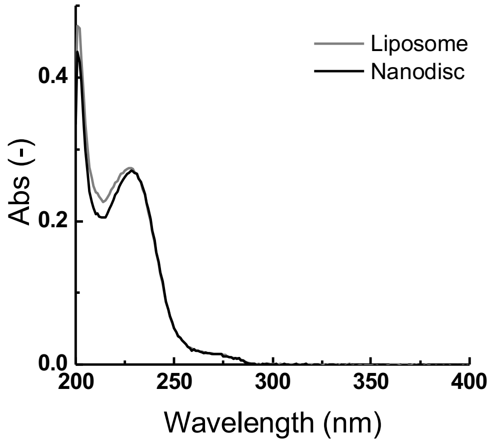

Fig. S10. The absorption spectrum of PTX loaded into liposomes and nanodiscs. [DPPC] = 316.8 μmol·L^{-1}, PTX 3 mol%, [DPPC] / [Polymer] = 8 for the nanodisc sample, the spectra were acquired by the dilution of each sample with 100 times larger volume of methanol to eliminate the background originating in the lipid aggregate in water.

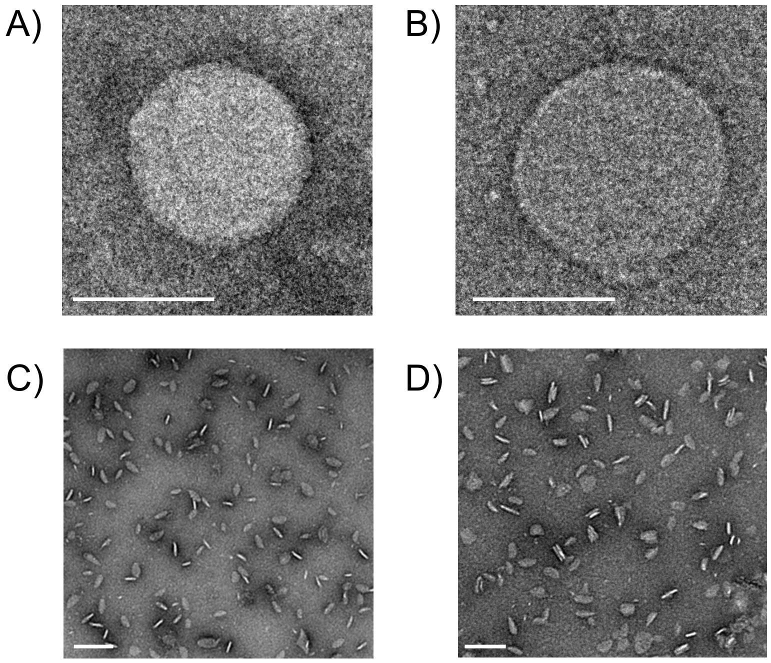

Fig. S11. TEM images for (A) DPPC liposome, (B) PTX-loaded DPPC liposome, (C) lipid nanodisc, and (D) PTX-loaded lipid nanodisc. [DPPC] / [Polymer] = 8 for nanodisc samples, [DPPC] = 1 mmol·L^{-1}, [PTX] = 30 μmol·L^{-1}, in PBS (pH = 7.4, [NaCl] = 150 mmol·L^{-1}), stained with phosphotungstic acid, scale bar = 100 nm.

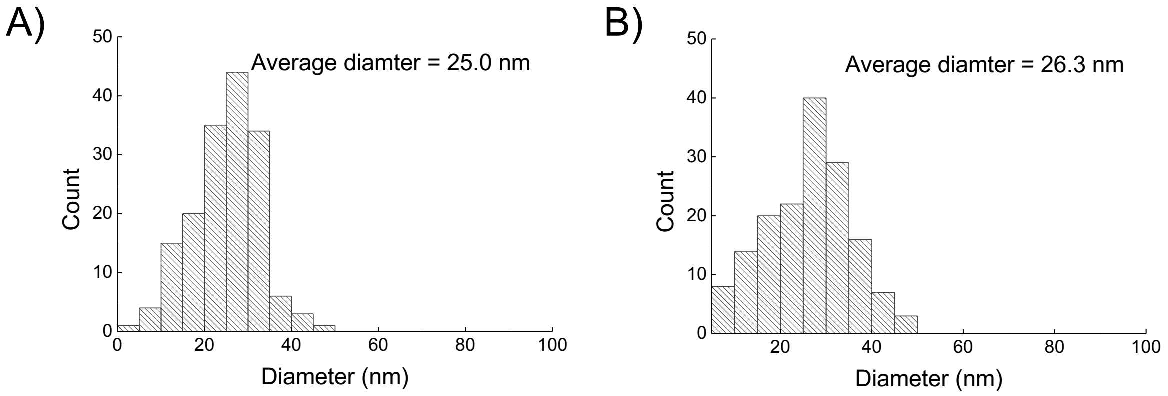

Fig. S12. Size distribution of nanodiscs estimated by the TEM image analysis. [DPPC] / [Polymer] = 8, [DPPC] = 1 mmol·L^{-1}, [PTX] = 30 μmol·L^{-1}, in PBS (pH = 7.4, [NaCl] = 150 mmol·L^{-1}). Each sample was counted over 100 nanodiscs using an Image J software.

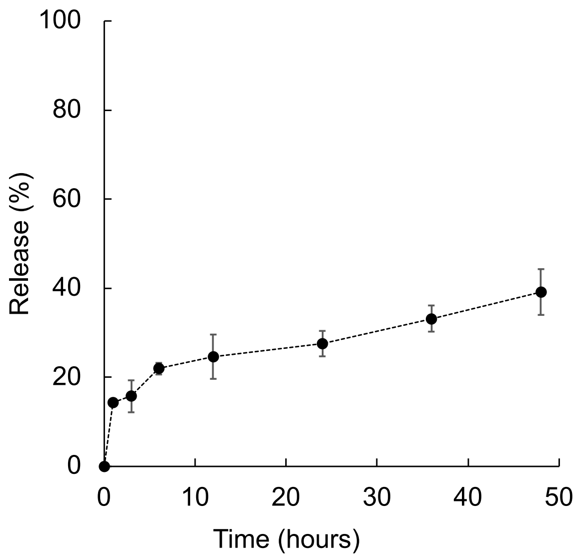

Fig. S13. Time-course of PTX release from nanodiscs evaluated by dialysis. 800 μL of the nanodiscs sample was introduced to the dialysis tube. [DPPC] / [Polymer] = 8, [DPPC] = 3.9 mmol·L^{-1}, [PTX] = 118 μmol·L^{-1}, in PBS (pH = 7.4, [NaCl] = 150 mmol·L^{-1}). The nanodisc sample was dialysed in 1 L of PBS buffer.

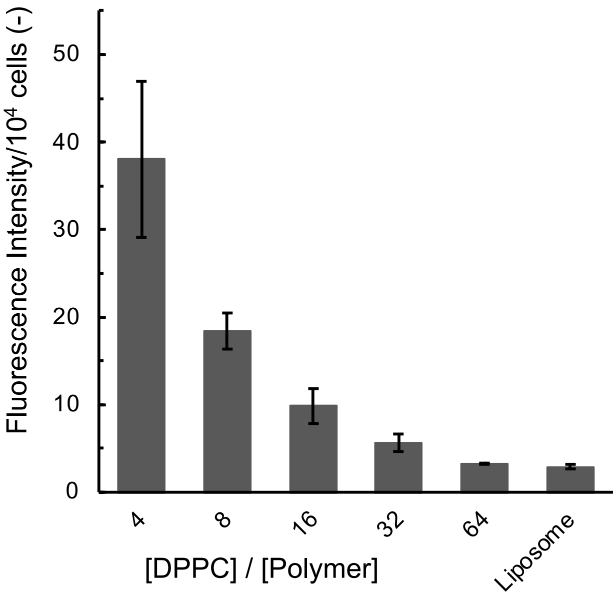

Fig. S14. Effect of [DPPC] / [Polymer] ratio on the internalisation of nanodisc into HeLa cells determined by fluorescence measurement. [DPPC]= 1.56 μmol·L^{-1}, HeLa cells were exposed to nanodiscs or liposomes at 37 °C for 48 hours.
